# Cellular immunity in children with successful immunoprophylactic treatment for mother-to-child transmission of hepatitis B virus

**DOI:** 10.1186/1471-2334-10-103

**Published:** 2010-04-28

**Authors:** Haruki Komatsu, Ayano Inui, Tsuyoshi Sogo, Eitaro Hiejima, Akihiko Tateno, Paul Klenerman, Tomoo Fujisawa

**Affiliations:** 1 Department of Pediatrics, Yokohama Eastern Hospital, 3-6-1 Shimosueyoshi Tsurumi Yokohama, Kanagawa 230-0012, Japan; 2 Department of Pediatrics, Sakura Medical Center, Toho University 564-1 Shimoshizu Sakura, Chiba 285-8741, Japan; 3 Nuffield Department of Medicine, Peter Medawar Building for Pathogen Research, University of Oxford, South Parks Road, Oxford, OX1 3SY, UK

## Abstract

**Background:**

The administration of hepatitis B immunoglobulin followed by hepatitis B vaccine can result in a protective efficacy of almost 90% in mother-to-child transmission of hepatitis B virus (HBV). However, little is known about immunity against HBV infection in children after immunoprophylactic treatment. We tried to assess the association between T-cell responses and viremia in children after successful prophylactic treatment.

**Methods:**

Thirteen children and their 8 HBV carrier mothers (8 families), who were positive for human leukocyte antigen (HLA)-A24, were enrolled in this study. All of the 13 children received immunoprophylactic treatment and became negative for hepatitis B surface antigen (HBsAg) after birth. HBV-specific cytotoxic T lymphocyte (CTL) responses were evaluated using IFNγ - enzyme-linked immunosorbent spot (ELISPOT) and major histocompatibility complex class I peptide pentamer assays. Serum HBV DNA was measured by real-time PCR.

**Results:**

Significant HBV-specific T-cell responses were detected in 2 (15%) of the 13 children by ELISPOT. However, the frequency of HLA-A24-HBV-specific CTLs was very low in both HBV carrier mothers and children using pentamers. Of the 13 children, 4 (31%) were positive for serum HBV DNA. However, the levels of serum HBV DNA were 100 copies/ml or less. One of the 2 children in whom significant HBV-specific CTL responses were detectable was positive for serum HBV DNA.

**Conclusions:**

HBV core and polymerase-specific T-cell responses were detected and a low-dose viremia was observed in children after successful immunoprophylaxis treatment. Although the presence of viremia was not related to HBV-specific T-cell responses, CTLs might play a role in the control of HBV infection in children born to HBsAg-positive mothers after immunoprophylactic treatment.

## Background

Worldwide, hepatitis B virus (HBV) is a common cause of liver disease. An estimated 350 million persons are chronically infected with HBV, and these individuals have a 15-25% risk of dying from HBV-related disease, including liver cirrhosis, hepatic decompensation, and hepatocellular carcinoma [1,2]. HBV is 100 times more infectious than human immunodeficiency virus and is transmitted by percutaneous or mucosal exposure to infected blood or other body fluids. Perinatal transmission, household contact, sexual contact, blood transfusion, and unsterilized injection are known as common routes of HBV transmission. The risk of mother-to-child transmission is 5-20% if the mother is positive for hepatitis B surface antigen (HBsAg) alone, but 90% if the mother is positive for hepatitis B e antigen (HBeAg) [3]. To prevent mother-to-child transmission at or around birth, hepatitis B immunoglobulin (HBIG) is administrated for newborns born to HBsAg-positive mothers within 12 hr after birth combined with a three-dose series hepatitis B vaccine in many countries, including Japan [4,5]. HBIG has high levels of antibodies to HBsAg (anti-HBs), which are neutralizing antibodies against HBV. HBIG is immediately effective and protective for a few months after birth. However, the levels of anti-HBs decrease over time. Therefore, active vaccination is required to sustain sufficient levels of anti-HBs to protect young infants from HBV infection. This combination strategy can show a protective efficacy of almost 90% and results in fewer than 5% of infants becoming HBV carriers [6-8].

Little is known about immunity from HBV infection in children after successful immunoprophylactic treatment, resulting in several questions about immunity post-vaccination. For example, it remains controversial whether the appearance of anti-HBs in children born to HBsAg-positive mothers implies complete protection from HBV infection after birth. Previous studies showed that serum HBV DNA was detected by polymerase chain reaction (PCR) in children born to HBsAg-positive mothers even after anti-HBs were induced by hepatitis B vaccine [9,10]. These findings suggested that children born to HBsAg-positive mothers have a risk of becoming HBV carriers even if immunoprophylactic treatment was successfully administered. Although the levels of serum HBV DNA are low in these anti-HBs-positive children after immunoprophylactic treatment, it is nevertheless a concern that reactivation of HBV replication could occur if these children receive immunosuppressive therapy.

In addition, the responses of HBV-specific cytotoxic T lymphocytes (CTLs) have never been evaluated in children after prophylactic treatment. HBV-specific CTLs play a major role in the control of HBV infection [11]. Because hepatitis B vaccine is derived from surface protein, theoretically Th2 cytokines associated with helper T lymphocytes are produced in response to vaccination [12]. To stimulate major histocompatibility complex (MHC) Class I restricted CD8+ CTLs, endogenous peptides processing and presentation is required. Although HBs peptide-specific CTLs can be induced by hepatitis B vaccine, whether CTL responses to other peptides derived from core and polymerase regions are primed remains unclear. There is, therefore, a possibility that HBV-core protein or -polymerase protein-specific CTLs would be detectable in children born to HBsAg-positive mothers if these children were exposed to HBV transiently or persistently at or after birth.

The aim of this study was to clarify the association between HBV-specific CTL responses and HBV viremia in children born to HBsAg-positive mothers after successful immunoprophylactic treatment. HBV-specific CTL responses were evaluated using enzyme-linked immunosorbent spot (ELISPOT) and MHC Class I peptide pentamer assays. Because human leukocyte antigen (HLA)-A24 is the most common HLA type in Japan, HBV-specific HLA-A24-restricted CTL epitopes were used for assays. Serum HBV DNA was measured by real-time PCR, which is a powerful tool for the detection of HBV DNA. Our data suggest that low-level viremia may occur and that this may be associated with priming of CTL responses even after apparently successful immunoprophylaxis.

## Methods

### Patients

**Figure 1:**
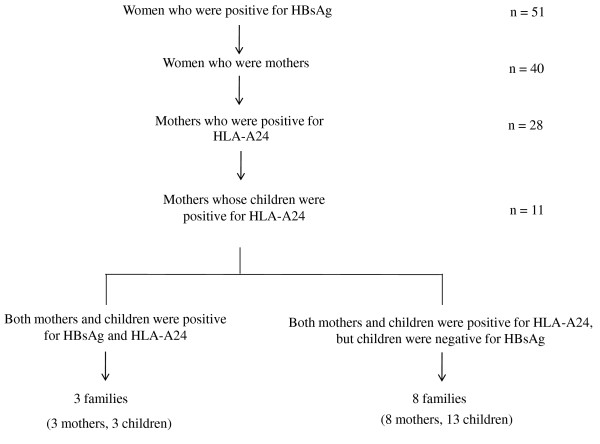
**Subject enrollment**.

This cross sectional study was approved by the Ethics Committee. Informed consent for study participation was obtained from the patients or their parents. Between 2002 and 2007, fifty-one women [HBeAg positive, n = 18; antibodies to hepatitis B e antigen (anti-HBe) positive, n = 33] [age range: 18-51 years; mean age ± SD: 34.4 ± 7.7 years] with chronic hepatitis B were followed in our department (Figure 1). All of the women were positive for HBsAg and negative for anti-HBs. None of them had received any anti-viral treatment before, during, or after delivery. Of the 51 women with chronic hepatitis B infection, 40 were mothers (HBeAg positive, n = 15; anti-HBe positive, n = 25). HLA typing using flow cytometry was performed in the 40 mothers, of whom 28 (HBeAg positive, n = 11; anti-HBe positive, n = 17) were positive for HLA-A24. Of the 28 HLA-A24-positive mothers, 25 (HBeAg positive, n = 10; anti-HBe positive, n = 15) were followed with their children in our department. The 25 mothers had 33 children (male/female = 16/17; age range: 0-22 years; mean age ± SD: 5.3 ± 4.4 years). Of the 33 children, 7 children were positive for HBsAg and the remaining 26 children were negative for HBsAg. Of the 33 children, 16 were positive for HLA-A24. Of the 16 children, 3 were chronically infected with HBV. These 16 HLA-A24-positive children were born from 11 of the 25 HLA-A24-positive mothers. Therefore, both mothers and children were positive for HLA-A24 in 11 families. Of the 11 families, 8 had 13 children who were negative for HBsAg. The 13 children (male/female = 6/7; age range: 0-13 years; mean age ± SD: 5.4 ± 3.4 years) and their 8 HBV carrier mothers (HBeAg positive, n = 2; anti-HBe positive, n = 6, age range: 22-43 years; mean age ± SD: 31.3 ± 7.6 years) were enrolled in this study (Figure 1). During the follow-up period, blood samples were collected from all patients.

### Monoclonal antibodies and synthetic peptides

Anti-HLA-A23, 24 monoclonal antibodies (mAbs) were purchased from One Lambda (Canoga Park, CA, USA). Goat anti-mouse immunoglobulin (IgG)-FITC Abs were purchased from Immunotech (Marseille, France). The HLA-A*2402 restricted HBV-specific CTL epitope core 117-125 (EYLVSFGVW) and HLA-A*2402-restricted HBV-specific CTL epitope Pol 756-764 (KYTSFPWLL) were previously identified [13]. These peptides were purchased from Nippi Research Institute of Biomatrix (Tokyo, Japan). Peptide purity was determined by mass spectrometry and high-pressure liquid chromatography.

### HLA typing

Detection of HLA-A24 was performed by flow cytometry using the HLA-A23, 24 mAbs as we described previously [14]. Because it is well established that HLA-A23 antigen is very rarely detected in Japanese populations [15,16], the HLA-A23, 24-positive subjects were considered to be positive for HLA-A24. In brief, 200 μL of EDTA or heparinized whole blood was stained with HLA-A23, 24 mAb for 20 minutes at 4°C. Whole blood stained with anti-HLA-A23, 24 mAb was washed with PBS. After incubation for 20 minutes at 4°C with FITC-labeled mouse IgG, the blood was washed again with PBS and lysed using FACS lysis solution (Becton Dickinson Sciences, San Jose, CA, USA). Samples were analyzed with a FACS Calibur machine (BD Bioscience, Becton Dickinson) using CellQuest software (BD Bioscience), after compensation was checked using freshly stained peripheral blood mononuclear cells (PBMCs).

### Pentamer assay

PBMCs were isolated from heparinized venous blood by density gradient sedimentation using Ficoll-Hypaque (Lymphoprep; Axis Shield, Oslo, Norway). PBMCs were stained with R-phycoerythrin (PE)-labeled Pro5 MHC Pentamer HLA-A*2402/EYLVSFGVW and HLA-A*2402/KYTSFPWLL (Proimmune, Oxford, UK) according to the manufacturer's instructions. In brief, 10 μL of labeled Pentamer was added to a million PBMCs and mixed by pipetting. Samples were incubated at room temperature for 10 minutes, shielded from the light. Cells were washed with 2 mL of PBS, and then 1 μL of anti-CD8 FITC was added to the samples, which were then mixed by pipetting. The samples were incubated at 4°C for 20 minutes, shielded from the light, and then the cells were washed twice with PBS. Cells were stored in Cell Fix buffer (Becton Dickinson Immunocytometry Systems) at 4°C until analysis. Samples were analyzed with a FACS Calibur using CellQuest software, after compensation was checked using freshly stained PBMCs. On the basis of a previous study, 0.04% of total CD8-positive cells were considered to show non-specific binding in HBV infection [17].

### ELISPOT assay

PBMCs were isolated from heparinized venous blood by density gradient sedimentation using Ficoll-Hypaque (Lymphoprep; Axis Shield, Oslo Norway). Fresh PBMCs were plated in 96-well polyvinylidene plates (Milipore, Bedford, MA, USA) that had been precoated with 0.5 μg/ml anti-IFN-γ-mAb (Mabtech, Stockholm, Sweden). The peptides were added in a volume of 10 ul, and then PBMCs were added at 50,000-100,000 cells/well in a volume of 190 μl. The final concentration of core 117-125 (EYLVSFGVW) and pol 756-764 (KYTSFPWLL) was 5 μM. The plates were incubated overnight at 37°C in 5% CO_2 _atmosphere and washed in PBS before the addition of the second biotinylated anti-IFN-γ-mAb (Mabtech) at 0.5 μg/ml, followed by incubation at room temperature for 100 min. After the plates were washed, streptavidin-conjugated alkaline phosphatase (Mabtech) was added at room temperature, and the samples sat for 40 minutes. Individual cytokine-producing cells were detected as dark spots after a 20-minute reaction with 5-bromo-4-chloro-3-indolyl phosphate and nitro blue tetrazolium using an alkaline phosphatase-conjugated substrate (Bio-Rad Laboratories, Hercules, CA, USA). The number of specific T cells was calculated by subtracting the negative control values and expressed as the number of spot-forming units per 10^6 ^PBMCs. Wells were considered to exhibit a significant HBV-specific T-cell response if they had values of least twice as great as the background value and the number of spots was more than 50.

### Real-time PCR assay

To evaluate the relation between HBV-specific T-cell responses and viremia, we examined whether HBV viremia was present in HBsAg-negative children. Real-time PCR is a sensitive method for detecting HBV DNA. We used the QIAamp UltraSens Virus DNA kit (QIAGEN, Valencia, CA, USA) to extract virus DNA according to the manufacturer's instructions. To enhance the detection, HBV DNA was extracted from 1 mL of serum and extracted DNA was concentrated. The extracted DNA was dissolved in 60 μL of elution buffer, and 10 μL of this solution was used for real-time PCR. Quantification of HBV DNA levels was performed using the genotype-independent real-time PCR method described previously [18]. In brief, PCR was performed on a 50-μL reaction mixture containing 25 μL TaqMan Universal PCR master mix (Applied Biosystems, Foster City, CA, USA) with 0.2 mM primers, 0.1 mM probes, and 10 μL extracted DNA. The PCR program consisted of an initial pre-cycle incubation at 50°C for 2 min and 95°C for 10 min, followed by 55 cycles of 95°C for 15 s and 60°C for 1 min. The PCR assay was performed in a MX3000P machine (Stratagene), and the results were analyzed with MxPro software (version 3.0). This assay has a quantitative detection limit of 80 copies/mL. Therefore, this in-house PCR was as sensitive as commercially available real-time PCR such as the COBAS TaqMan HBV test (Roche).

### Statistical analysis

The Chi-square test and Fisher's exact tests were used to compare frequencies. Results are presented as the mean ± SD. Means were compared using Student's *t*-test or the Mann Whitney test. A *P*-value of .05 or less was considered to indicate statistical significance.

## Results

### Characteristics of children and HBV carrier mothers

To evaluate the immunity in children with successful immunoprophylactic treatment, we classified the children into two groups according to their HBsAg status. One group was the children who received immunoprophylactic treatment (HBIG combined with a 3-dose series hepatitis B vaccine) after birth and remained negative for HBsAg (13 children from 8 families) (Table 1). The other group was the children who were positive for HBsAg (3 children from 3 families) (Table 2). Of the 3 HBsAg-positive children, one (C10) did not receive immunoprophylactic treatment and became an HBV carrier. Despite immunoprophylactic treatment, the remaining two children in this group also became HBV carriers.

**Table 1:** Patient characteristics, IFN-γ enzyme-linked immunosorbent spot, and serum HBV DNA levels of children with successful prophylactic treatment and their mothers.

Patient ID*	Age (years)	Gender	Levels of serum hepatitis B surface antibodies (mIU/mL)	Hepatitis B e antigen status	Hepatitis B core antibody status	Levels of serum ALT (IU/L)	† core 117-125 (spots/10^6 ^PBMCs)	† pol 756-764 (spots/10^6 ^PBMCs)	Levels of serum hepatitis B virus DNA (copies/mL)
M1	22			Pos	Pos	18	14.1	17.6	> 10^8.8^
C1	2	Female	84.7		Neg	16	0	5.0	100
									
M2	28			Neg	Pos	13	0	0	10^4.0^
C2	2	Male	40.6		Neg	17	0	8.9	80
									
M3	43			Neg	Pos	10	4.5	0.9	10^3.2^
C3a	11	Female	16.2		Neg	8	0	6.5	Neg
C3b	6	Male	172.1		Pos	17	0	0	Neg
									
M4	32			Neg	Pos	11	30.8	47.3	10^3.1^
C4	6	Male	28.2		Neg	15	13.9	94.4	Neg
									
M5	34			Neg	Pos	37	0	0	10^3.0^
C5a	8	Female	118.9		Neg	20	12.5	0	Neg
C5b	6	Female	63.4		Neg	13	68.0	114.3	80
									
M6	32			Neg	Pos	17	48.2	8.2	10^3.7^
C6a	6	Female	6.3		Neg	11	0	0	Neg
C6b	4	Female	<5		Neg	12	40.0	0	Neg
C6c	2	Male	<5		Neg	11	2.9	29.4	Neg
									
M7	40			Pos	Pos	14	9.8	18.5	10^8.1^
C7a	13	Male	<5		Neg	18	0	0	Neg
C7b	4	Female	34.2		Neg	11	0	0	Neg
									
M8	22			Neg	Pos	15	0	0	10^4.3^
C8	0	Male	252.9		Pos	17	29.1	0	80

* M and C indicate mother and child, respectively. Number indicates family ID. † More than 50 spots/10^6 ^PBMCs were defined as a significant HBV-specific T-cell response. PBMCs, peripheral blood mononuclear cells. Pos, positive Neg, negative

**Table 2:** Patient characteristics, IFN-γe nzyme-linked immunosorbent spot, and serum HBV DNA levels of hepatitis B surface antigen-positive children and their mothers.

Patient ID*	Age (years)	Gender	Levels of serum ALT (IU/L)	Hepatitis B e antigen status	† core 117-125 (Spots/10^6 ^PBMCs)	† pol 756-764 (Spots/10^6 ^PBMCs)	Levels of serum hepatitis B virus DNA (copies/mL)
M9	33		18	Pos	12.6	53.9	>10^8.8^
C9	3	Male	33	Pos	12.8	5.7	10^8.2^
							
M10	47		21	Pos	12.8	30.8	10^8.3^
C10	22	Female	30	Pos	20.7	26.2	10^8.6^
							
M11	32		17	Pos	22.5	48.4	10^8.6^
C11	4	Female	16	Pos	7.0	5.3	10^8.7^

* M and C indicate mother and child, respectively. Number indicates family ID. † More than 50 spots/10^6 ^PBMCs were defined as a significant HBV-specific T-cell response. PBMCs, peripheral blood mononuclear cells. Pos, positive Neg, negative

Characteristics of HBsAg-negative children and their mothers are shown in Table 1. Among the parents of children with successful immunoprophylactic treatment, 2 mothers were positive for HBeAg and the remaining 6 were negative for HBeAg. The levels of serum alanine aminotransferase (ALT) (normal range: <35 IU/L) were slightly elevated in one mother (M5). On the other hand, the levels of serum ALT were normal in all children with successful immunoprophylactic treatment. Although 10 mIU/mL or more was considered a protective level of serum anti-HBs, the level of serum anti-HBs was less than 10 mIU/mL in 4 children (C6a, C6b, C6c, and C7a). Antibodies to hepatitis B core (anti-HBc) are considered to be a marker of present or past infection. Two children (C3b and C8) were positive for anti-HBc. Because one child was a six-month-old baby, it was thought that his maternal anti-HBc could cross the placenta at birth.

Table 2 shows the characteristics of HBsAg-positive children and mothers. All of the children and mothers were positive for HBeAg. The levels of serum ALT were normal in all of these children and mothers.

### ELISPOT assays

We evaluated the function of HBV-specific T-cells in HBsAg-negative children (those who had received successful prophylactic treatment for mother-to-child transmission), HBsAg-positive children, and HBsAg-positive carrier mothers. IFNγ-ELISPOT assays were performed in both children and mothers. The results of ELISPOT assays in the group of HBsAg-negative children (n = 13)/HBsAg-positive mothers (n = 8) and HBsAg-positive children (n = 3)/HBsAg-positive mothers (n = 3) are shown in Tables 1 and 2, respectively. Surprisingly, a significant HBV-specific T-cell response (> 50 spots/10^6 ^PBMCs) was detectable in 2 of 13 HBsAg-negative children (C4: pol 756-764; C5b: core 117-125, pol 756-764). However, anti-HBc antibodies were not detectable in the 2 children who showed significant HBV-specific T-cell responses.

No significant HBV-specific T-cell response was detected in the mothers of HBsAg-negative children. Likewise, none of the HBsAg-positive children had a significant response (Table 2), but one HBV carrier mother showed a significant response (M9; pol 756-764) (Table 2). Although there was no significant difference in the frequency of HBV-specific T-cell response to the two HBV-derived peptides between HBsAg-negative children and HBsAg-positive children (Table 3), these findings suggested that HBsAg-negative children who received prophylactic treatment have a potential to produce HBV-specific T-cell responses.

**Table 3:** The frequency of children positive for IFN-γ enzyme-linked immunosorbent spot assay HLA-A24 hepatitis B virus-specific cytotoxic T-cell epitope.

	HLA-A24 hepatitis B virus-specific cytotoxic T-cell epitope			
	
	core 117-125 No. of children (%)		pol 756-764 No. of children (%)	
Hepatitis B surface antigen-positive children n = 3	0 (0)	\	0 (0)	\
		P = 0.41		P = 0.81
Hepatitis B surface antigen-negative children n = 13	1 (7.7)	/	2 (15.4)	/

### HBV pentamer analysis

HBV-specific CTLs play a crucial role in both virus clearance and the pathogenesis of hepatic cell injury. In particular, CTLs show a stronger response in cases of acute infection than in patients with chronic infection. MHC Class I peptide complexes (pentamers) can detect circulating HBV-specific CD8+ T cells. We performed pentamer staining to detect HBV-specific CD8+ T cells in HBsAg-negative children (n = 10) and their mothers (n = 5) (Table 4). None of the HBsAg-negative children showed a high frequency of pentamer-positive cells (Pentamer/CD8, core 117-125: 0.003 ± 0.005%, pol 756-764: 0.002 ± 0.002%). Similarly, HBsAg carrier mothers did not show a high frequency of HBV-specific CD8+ T cells (Pentamer/CD8, core 117-125: 0.004 ± 0.005%, pol 756-764: 0.001 ± 0.002%). Because there was no study participant who showed more than 0.04% in frequency of HBV-specific CD8+ T cells in this study, neither the HBsAg-negative children nor their mothers were classified as showing a positive response in this study. However, we did not have any positive controls. Therefore, further studies are required to interpret these results appropriately.

**Table 4:** Pentamer assay in the hepatitis B virus carrier mothers and hepatitis B surface antigen-negative children after successful prophylactic treatment of mother-to-child transmission.

	Pentamer/CD8 mean ± SD (%)	
	
	core 117-125	pol 756-764
Mother (n = 5)	0.004 ± 0.005	0.001 ± 0.002
Children (n = 13)	0.003 ± 0.005	0.002 ± 0.002

### Serum HBV DNA, serum HBsAb, and HBeAg status of mothers

The levels of serum HBV DNA are shown in Table 1. Of the 13 HBsAg-negative children, 4 (31%) were positive for serum HBV DNA. However, the levels of serum HBV DNA were between 100 and 80 copies/mL. All of the children with HBV viremia were positive for anti-HBs. Although all of the 13 HBsAg-negative children received prophylactic treatment, anti-HBs were not detectable in 3 of these children (23%). One of the children with viremia (C1) was born to an HBeAg-positive mother, and the remaining 3 children with viremia (C2, C5b, and C8) were born to HBeAg-negative mothers. Of 2 children who showed a significant response to HBV peptides in ELISPOT assay, one child (C5b) was positive for serum HBV DNA. Therefore, there was no association between the detection of serum HBV DNA and HBV-specific T-cell responses.

## Discussion

Newborns born to HBsAg-positive mothers are usually exposed to HBV around delivery, except those who contract intra-uterine infection. The administration of HBIG containing neutralizing antibodies can prevent newborns from HBV infection for a few months after birth. Active hepatitis B vaccine following HBIG administration induces active immunity and stimulates the production of sustained neutralizing antibodies against HBV in newborns. However, the overall immune response induced by both HBIG and hepatitis B vaccine in the prevention of mother-to-child transmission remains unclear. Although a small proportion of newborns treated with HBIG combined with hepatitis B vaccine become positive for HBV DNA from cord blood or peripheral blood transiently at or after birth, they eventually show no viremia [9,10,19]. In contrast, a proportion of newborns receiving HBIG combined with hepatitis vaccine become positive for anti-HBs, but HBV viremia persists after birth [10]. These observations suggest that newborns can be suffering from HBV viremia transiently or for several years after birth, despite having received immunoprophylactic treatment. Our goal was to assess the association between T-cell responses and viremia in children after successful prophylactic treatment.

HBV viremia stimulates host cellular immunity, and CTLs can be involved in viral control [11,17]. The induction of HBV-core or polymerase-specific CTL indicates that HBV infects host cells and that HBV proteins presented on the surface of the host cells are recognized as targets by the host. In this study, 2 (15%) of 13 children who were negative for HBsAg showed significant responses in IFN-γ ELISPOT against HBV peptides. Because HBV-specific peptides were derived for core and polymerase protein in this study, these findings suggested that HBV-specific T-cells were primed due not to hepatitis B vaccine but to the infection with HBV. In addition, one of the 2 children in whom HBV-specific T-cell responses were detectable was positive for serum HBV DNA. Although the levels of serum HBV DNA were low, viremia persisted despite HBV-specific T-cells and anti-HBs remaining detectable. These findings were similar to those in patients who suffered from acute HBV infection [20,21]. In acute HBV infection, clinical recovery does not imply the complete clearance of serum or intrahepatic HBV DNA. The authors of previous studies reported that HBV viremia persisted and anti-viral T-cell responses were detectable for several years after resolution of acute hepatitis B [20-22]. The levels of serum HBV DNA are usually low after acute hepatitis B.

In animal models of acute hepatitis B, functional T-cell responses were generated after infection using high doses of inoculated virus [23]. The results of one previous study suggested that exposure to a low viral load allows persistent viremia without priming functional T-cells [24]. Similarly, detection of functional T-cells might be closely related to the load of HBV to which children are exposed before, during, and after prophylactic treatment. However, the mothers of the 2 children in whom HBV-specific T-cells were detected were negative for HBeAg. Because HBeAg-negative mothers usually have a low viral load (Table 1), viral exposure alone might not explain the induction of HBV-specific T-cells in children. Some studies suggested that HBeAg might induce T cell tolerance in vertical transmission [25]. Therefore, viral genome mutations such as precore mutants, which are unable to secrete HBeAg, could be associated with the outcome of mother-to-child transmission [26]. In C4, ELISPOT showed a positive result for polymerase protein but not core protein. This finding was consistent with that of a previous study, in which some patients showed positive results for both peptides and others showed positive results for only one peptide in acute HBV infection [17]. Although CTL responses are restricted with the HLA type, CTL responses to viral epitopes were different in each individual.

Various studies have been performed using PCR to evaluate the efficacy of immunization against HBV in infants born to HBV carrier mothers [9,10]. In a previous study, 2 of 29 (7%) children who received HBIG and hepatitis B vaccine and became negative for HBsAg and positive for anti-HBs were positive for serum HBV DNA [9]. Of the 2 children, one was free of HBV viremia 2 years later. The other child became positive for HBsAg and negative for anti-HBs later, because the mutation in the "a" determinant of the S region occured. In another study, serum HBV DNA was detected at 4-6 years after birth by PCR in 14 of 94 (14%) children who received prophylactic treatment [10]. Of the 14 children, 7 were negative for HBsAg and positive for anti-HBs. Three of the 7 children showed a high viral load (> 10^5 ^copies/mL), and the remaining 4 children showed a low viral load (< 200 copies/mL). In the present study, real-time PCR was adopted to detect serum HBV DNA, as real-time PCR can be more sensitive than conventional PCR. In addition, serum HBV DNA was quantified more accurately. Serum HBV DNA was detected in 4 of 13 (31%) children in our study, but the levels of HBV DNA were between 100 and 80 copies/mL. The prevalence rate of HBV viremia in this study was higher than that in previous studies [9,10]. The difference in the prevalence was presumably caused by the difference in the detection limit of each PCR method. Anti-HBc were detected in one of the 4 children with viremia. However, the child was a 6-month-old boy. Probably, maternal anti-HBc were transferred to the child through the placenta at birth [27]. Of the 4 children with viremia, 3 were born to HBeAg-negative mothers. In general, babies born to HBeAg-negative mothers are unlikely to become HBV carriers, even if immunoprophylactic treatment is not given. We cannot answer the question why 3 of 4 children with viremia were born to HBeAg-negative mothers. In a future study, the prevalence of viremia in a large number of children who received successful prophylactic treatment should be assessed.

Although escape mutants have been associated with persistence of serum HBV DNA after acute hepatitis B, the appearance of an escape mutant is uncommon in acute hepatitis B. In a previous study, variants in the S region were detected in 2 of 14 children who received prophylactic treatment [10]. However, the 2 children were positive for HBsAg, negative for anti-HBs antibodies, and infected with a high viral load (>10^8 ^copies/mL). The previous study results suggested that the majority of patients with viremia were infected with wild-type HBV after prophylactic treatment. Although we did not sequence the S region, the mechanism of persistent viremia in children after the appearance of anti-HBs antibody might be different from that in children infected with escape variants. The level of serum anti-HBs antibodies considered protective is >10 mIU/mL [28]. In the present study, the level of serum anti-HBsAb was < 10 mIU/mL in 4 of the 13 children who were negative for HBsAg after prophylactic treatment. Although it is controversial whether booster vaccination is required to maintain adequate levels of anti-HBs antibodies [28-30], all of the 4 children were negative for serum HBV DNA. All of these children had levels of anti-HBs antibodies >10 mIU/mL at 1 year after birth. However, the levels of anti-HBs antibodies decreased to < 10 mIU/mL over time. None of the 4 children showed a significant HBV-specific T-cell response. Further studies are needed to evaluate the role of HBV-specific T-cell responses in children who lose anti-HBs after prophylactic treatment.

The results of this study raise questions about the long-term clinical impact of a low-dose HBV infection in children. For instance, children with a low-dose infection could suffer from hepatocelluar carcinoma in the future. In addition, the new emerging biological therapies for immunomodulation, including tumor necrosis factor inhibitors and anti-B cell agents such as Rituximab, will induce reactivation of HBV with a potentially fatal outcome in adult patients with chronic HBV infection or occult HBV infection [31,32]. Because this study was cross-sectional, we could not answer these questions. Longitudinal studies in children receiving prophylactic treatment are necessary to properly assess these issues and answer these questions.

## Conclusion

In conclusion, HBV core and polymerase-specific T-cell responses were detectable in children after the appearance of anti-HBs antibodies, although rarely. Moreover, a low-level viremia was observed in nearly 1 in 3 children. Although the presence of a low-level viremia was not related to HBV-specific T-cell responses, our study results showed that CTLs might play a role in the control of HBV infection in children born to HBsAg-positive mothers after immunoprophylactic treatment. Because the number of subjects in the present study was small, additional studies are required to evaluate virological and immunological issues, including the role of CTLs in children after immunoprophylactic treatment.

## Abbreviations

Anti-HBe: antibodies to hepatitis B e antigen; Anti-HBs: antibodies to hepatitis B surface antigen; CTLs: cytotoxic T lymphocytes; ELISPOT: enzyme-linked immunosorbent spot; HBeAg: hepatitis B e antigen; HBIG: hepatitis B immunoglobulin; HBsAg: hepatitis B surface antigen; HBV: hepatitis B virus; MHC: major histocompatibility complex; PBMCs: peripheral blood mononuclear cells.

## Competing interests

The authors declare that they have no competing interests.

## Authors' contributions

HK and PK contributed to the design of this study and drafted this manuscript. AI, TS, EH, AT, and TF participated in data collection and critically revised the manuscript. All the authors concurred with the submission and will take the responsibility for the manuscript.

## Pre-publication history

The pre-publication history for this paper can be accessed here:

http://www.biomedcentral.com/1471-2334/10/103/prepub
